# Narrative descriptions of miyo-mahcihoyān (physical, emotional, mental, and spiritual well-being) from a contemporary néhiyawak (Plains Cree) perspective

**DOI:** 10.1186/s13033-016-0086-2

**Published:** 2016-09-21

**Authors:** Holly Graham, Stephanie Martin

**Affiliations:** 1 College of Nursing, University of Saskatchewan, 4224 E-Wing, 104 Clinic Place, Saskatoon, SK Canada; 2 Educational Psychology and Special Education, University of Saskatchewan, Saskatoon, SK Canada

**Keywords:** Indigenous, Aboriginal, First nations, Plains Cree, Mental health perceptions, Determinants of health, Well-being, Narrative inquiry, Resilience

## Abstract

**Background:**

There are unequivocal health disparities, both physical and mental, between the Indigenous and non-Indigenous peoples of Canada.

**Methods:**

Utilizing narrative inquiry, 15 néhiyawak (Plains Cree people) between 18 and 71 years of age from Thunderchild First Nation were interviewed to explore what improved their mental health and well-being and what they needed to attain optimal mental health and well-being. By posing questions that focused on the positive, the strengths and resilience of the néhiyawak came to the forefront.

**Results:**

Narrative thematic analysis of interview data consistently revealed four overarching themes that highlighted what positively impacted néhiyawak mental health and well-being and their perceived needs to attain optimal mental health and well-being: relationships; spiritual beliefs and cultural practices; tānisīsi wāpahtaman pimātisiwin (worldview); and ēkwa ōhi kikwaya piko ka-ispayiki kīspin ka-nohtē-miyo-mahcihoyān (these are the things that need to happen if I want to be healthy). The néhiyawak in this study described holistic health determinants that correlate with the medicine wheel and the determinants of health, and described these holistic health determinants as making a positive difference to their mental health and as necessary for them to obtain optimal mental health and well-being.

**Conclusions:**

These results suggest that mental health programming and interventions should be harmonious with Indigenous culture; utilize a holistic approach that takes physical, emotional, mental, and spiritual well-being into consideration; and address the existing mental health disparities using the determinants of health as a framework, with an increased focus on the current socio-economic status of Indigenous peoples in Canada.

## Introduction

There is an abundance of literature that clearly describes the existing health disparities between Indigenous and non-Indigenous people in Canada. It is timely to collaborate respectfully with Indigenous peoples to ask them what contributes to their mental health and well-being. Having this first-hand knowledge is an important first step in mental health program planning and in delivering effective mental health care to Indigenous peoples.

The primary goal of this research project was to explore factors that improve the mental health and well-being of the *néhiyawak* from Thunderchild First Nation and what they perceived as necessary to attain optimal mental health and well-being. Secondly, the first author, an Indigenous woman, wanted to conduct research that was strength-based and would give voice to contemporary Indigenous (Plains Cree) people from her community of Thunderchild First Nation, Saskatchewan.

## Background

Indigenous peoples were historically autonomous, then colonized, and now, approximately 500 years later, continue to be disproportionately represented in most physical and mental health issues when compared to non-Indigenous people in Canada [3, 26, 60]. Indigenous people as a population do not have the same health status as other Canadians [3, 7, 8, 25–27, 60–62] and their socio-economic conditions are often cited as being similar to those in developing countries [26, 42].

Why does the health and mental status of Indigenous people continue to lag behind non-Indigenous people in Canada? This is an important question, and the answer is embedded within the Indigenous/non-Indigenous historical relationship, specifically colonization. This historical relationship is the fundamental root of the current health disparities experienced by Indigenous people on a daily basis [8, 30, 33, 42, 43, 62].

A range of epidemiological studies have documented high levels of mental health problems in many Canadian Aboriginal communities [2, 46, 53, 61]. Among other health disparities, Indigenous peoples have disproportionately higher rates of injury, trauma [7, 29], and suicide [21, 25, 29]. The First Nations Regional Health Survey [FNRHS] reported that, “in addition to death and disability, injuries (including those resulting from sexual violence) can lead to a variety of other health problems including depression, alcohol and substance abuse, eating and sleeping disorders, and HIV and other sexually transmitted diseases” ([14], p. 78). Injuries among First Nations adults are almost three times the Canadian average; almost one-third of these adults required treatment, which is twice the Canadian average. One in 20 reported they had suffered at least one instance of violence in the previous year. Injury is one of the leading causes of death, and is responsible for approximately one quarter of all deaths and over half the potential years of life lost [15]. Death from injury and poisonings is 2.9 times higher for Indigenous Peoples compared to the general Canadian population [24].

More recently, Khan [28] identified the following mental health challenges for Indigenous people: suicide, alcohol and drug use, and depression. Indigenous suicide rates are twice the national average [31], with Inuit suicide rates 6–11 times the Canadian average; depression is experienced twice the national average among Indigenous people; and 75 % of respondents reported alcohol use as a problem in their community [28]. First Nations communities participating in a national survey between 2008 and 2010 reported that alcohol and drug abuse were the number one challenge for community well-ness faced by on-reserve communities (82.6 % of respondents), followed by housing (70.7 %) and employment (65.9 %) [15].

In addition, as a result of the colonization process, Indigenous peoples have been subjected to a multitude of traumatic experiences [45, 62]. Unfortunately, contemporary Indigenous people continue to experience trauma either through neocolonial practices [62] or through injury [7]. Indigenous peoples have approximately a four-fold risk of severe trauma (physical) when compared to the non-Indigenous population [29].

According to Health Canada [25], suicide and self-inflicted injury are the leading causes of death for First Nations youth and adults up to 44 years of age. Karmali et al. [29] found that Aboriginal Canadians had a three times greater risk of traumatic suicide. Kirmayer et al. [32] identified risk factors that appear to be similar across the studies of Aboriginal youth suicide, including (a) male gender, (b) history of substance abuse (especially solvents or inhalants), (c) history of psychiatric problems, (d) parental history of substance abuse, or (e) physical abuse. According to Kirmayer et al. [31], suicide emerges from a complex interaction of biological, psychological, social, and cultural processes. They assert that “these factors influence the person from infancy onward, increasing resilience or making individuals more vulnerable to the effects of stress, conflict, violence, and loss. [In addition,] social, economic, cultural, and political factors may create predicaments that drive vulnerable individuals to suicidal behavior” (p. 33). Antone et al. [3] eloquently summarize, “by all measurements of the human condition, indigenous people lead in the statistics of suicide, alcoholism, family breakdown, substance abuse…they serve as direct indicators of the serious stress connected with being an indigenous person in today’s world” (p. 6).

Given the growing awareness of the many social and economic issues that have and continue to have an impact on First Nations communities, there has been increased urgency to develop and implement effective community interventions to address existing mental health issues.

## Methods

### Study design and sampling

Narrative inquiry was used to guide the research process, and thematic narrative analysis was used to analyze the data. Clandinin and Connelly [9] described narrative inquiry as “stories lived and told … a way of understanding experience” (p. 20) that allows all of us to learn. Narrative inquiry is culturally appropriate and congruent with the Plains Cree worldview because of its alignment with Indigenous epistemology [35, 51] and the centrality of the story [35].

A variety of mediums were used to inform the community members in Thunderchild First Nation of the research study. Posters were hung in the school, Band office, and the health clinic. Alongside the poster, there were recruitment letters for potential néhiyawak to take that included information on the nature and purpose of the research, criteria to participate, and contact information for both researchers. Some of the néhiyawak saw the primary researcher hanging the posters in the community, which led to conversations about the project, and they often expressed their interest to participate at that time. Snowball sampling [17] was utilized to obtain several participants for this study. When each interview was completed, the first author asked the participant if there was anyone else they knew who may interested in and appropriate for the study. If the néhiyawak could identify potential participants, they were asked to share the primary researcher’s contact information with the individual. This process ensured that the néhiyawak who were interested in participating could decide for themselves to contact the primary researcher, thereby increasing autonomy and some degree of anonymity.

Néhiyawak who participated in this study met the following criteria: (a) Band member of Thunderchild First Nation, living on or off reserve, (b) 18 years of age and over, and (c) interested in participating in this project. Within this study, Elders were: (a) Band members of Thunderchild First Nation, living on or off reserve, (b) greater than 50 years of age, (c) identified by Chief and Council or other members of the community, and (d) interested in participating in this project.

Fifteen néhiyawak agreed to participate in a semi-structured narrative interview. néhiyawak were between 18 and 71 years of age; seven were male and eight were female. Of these 15 participants, three were Elders: two female Elders were 62 and 70 years old, one male Elder was 62 years old. The néhiyawak level of education varied from grade 4 to completion of an undergraduate university degree. In addition, 13 of the 15 néhiyawak had a source of income either from being employed, receiving a pension, or living with their parents while attending high school.

### Narrative interviews

A semi-structured interview guide comprised of six questions (e.g., Tell me what mental health/wellness means to you? What is making a difference for you to be “mentally” healthy (if required ask: share an example)? How have you coped with the stressors in your life (if required ask: share an example)? Tell me what has helped you deal with the stressors in your life (if required ask: share an example)? Tell me what gives you hope (for the future/keeps you going) (if required ask: share an example)? Imagine yourself as mentally healthy/well as possible, what would have to happen for you to achieve that?) was used to broadly structure the interview process. The primary researcher ensured each participant had ample opportunity to share their stories in their own ways, in their own time.

The interviews began with a scripted prelude to ensure each *néhiyaw* received the same information about the process. After the participants answered the first question, the primary researcher shared two descriptions of mental health. The first description was from a Western paradigm and the second was from an Indigenous worldview. The script was: “The Canadian Mental Health Association [5] uses the following key characteristics to assess mental health: Ability to enjoy life (can you live in the moment and appreciate the ‘now’?); resilience (are you able to bounce back from hard times?); balance (can you recognize when you might be devoting too much time to one aspect, at the expense of others?); self-actualization (do you recognize and develop your strengths so that you can reach your full potential?); flexibility (do you feel, and express, a range of emotions? When problems arise, can you change your expectations—of life, others, and yourself—to solve the problem and feel better?)” (para. 5). Next, an Indigenous perspective of health [63] was shared: “Traditional American Indian health embodies a holistic health concept in which an individual has harmony with oneself, mind, body, and spirit; with others; and with his or her surroundings or environment” (p. 48).

The first author met with each participants two times. The first meeting at the health clinic was to answer their questions, obtain consent, and complete the audio-recorded interview. Two to three weeks later, a second interview was attended by most participants which provided an opportunity for them to review their transcript from the first interview and change any content if desired. For the néhiyawak who were unable to meet in person a second time at the health clinic, their second meeting occurred over the telephone.

### Thematic narrative analysis

In thematic narrative analysis, emphasis is on “what” is spoken and the content of speech is the exclusive focus ([50], p. 19). Each audio-taped interview was transcribed verbatim. The transcripts were coded with three different colours of text. Black represented the participant’s comments. Red represented the first author’s comments throughout the interview and her notes taken during the interview. Light blue represented the changes/additions made by the participant in the second interview (done during second meeting). Essentially, the participants’ responses were left intact as a narrative, with minor edits to the language in the narratives to “make it easily readable” ([50], p. 58). The primary researcher worked with a single interview at a time, isolating and organizing relevant events into a sequential biographical account. After the process had been completed for all interviews, the researcher identified the underlying assumptions in each account and named (coded) them. Particular cases were selected to illustrate general patterns [50]. Both researchers (Indigenous and non-Indigenous) reviewed and analyzed the data. Once the themes were identified, the findings were discussed with the Chief and Council and Health Board of the Thunderchild First Nation for feedback and input.

### Ethical approval

Historically the research conducted in Indigenous communities has not been a positive experience for Indigenous peoples, resulting in additional challenges for research [54, 57]. The historical conduct of research with Indigenous peoples has been described as, “Western knowledge, with its flagship of research, has often advanced into Indigenous Peoples’ communities with little regard for the notions of Indigenous worldviews and self-determination” [12]. Given the history and context in which research has been traditionally conducted in Indigenous communities, it was important for the project to be aligned with the changing philosophy and research practices being recommended and endorsed by Indigenous peoples [13] and the Canadian Institutes of Health Research, Natural Sciences and Engineering Research Council of Canada, and Social Sciences and Humanities Research Council of Canada, Tri-Council Policy Statement (TCPS2): Ethical Conduct for Research Involving Humans (2010). The TCPS 2—Chapter 9, provides a framework for the ethical conduct of research with Aboriginal peoples and encourages collaboration and authentic engagement and reciprocation between researchers and participants.

As part of the ethical preparation for the research, the primary researcher met with the Chief and Council three times to discuss the research project in advance, and once with the Health Director and health staff. Approval was obtained by the University of Saskatchewan Behavioural Research Ethics Board and the Chief and Council of the Thunderchild First Nation, via a Band Council Resolution (BCR).

### Reflexivity

Reflexivity acknowledges the potential for the interviewer’s subjectivity and personal bias to influence the interpretation of the data [10]. The primary author is a female member of Thunderchild First Nation. She kept an audit trail and personal journal. As well, she wrote her autobiographically oriented narrative in response to the research topic ([9], p. 41) prior to embarking on the project, and engaged the participants in member checking [37].

## Results

Analysis of the néhiyawak narrative interviews consistently revealed four overarching themes that highlighted what positively impacted their mental health and well-being, and what they need to attain optimal mental health and well-being: (a) relationships; (b) spiritual beliefs and cultural practices; (c) tānisīsi wāpahtaman pimātisiwin (worldview); and (d) ēkwa ōhi kikwaya piko ka-ispayiki kīspin ka-nohtē-miyo-mahcihoyān (these are the things that need to happen if I want to be healthy). An elaboration of these themes in the words of the néhiyawak follows.

### Relationships

The néhiyawak found that relationships met their emotional needs, increased their self-awareness, provided opportunities for personal growth, and gave them hope. The néhiyawak consistently described these relationships as improving their mental health and essential for optimal mental health and well-being. One very young lady described how the support she received from her friends, family, and coaches made a difference to her mental health and well-being. Being able to talk about her concerns with others gave her ideas and different perspectives on problem solving, “they tell me what they did at that time, so then I have an idea of how …I’d like to handle my situation.” One woman said helping others made a difference to her mental health and well-being. She had earned respect, felt needed, and enjoyed a sense of pride from helping others with their problems. She shared, “I help kids when they have problems…I give them a place to sleep and a place to eat… a lot of kids call me ‘mom’…it makes me feel proud; it makes me feel …wanted.”

One middle-aged woman spoke of both the negative and positive relationships in her life and her past experiences had impacted her mental health and increased her self-awareness. She shared how she had been abused while she was in foster care and when she was adopted her life drastically improved. Her foster mother’s “morals [and]…nagging…really had an impact on the way I think…mom’s always said, ‘life is tough, life is what you make it.’” When the néhiyawak were asked what gave them hope, what kept them going, their first response was generally related to their relationships. One middle-aged man spoke about his children giving him hope and keeping him going: “My kids give me hope. I don’t want my kids to see me as being a quitter or giving up.”

These relationships allowed them to express their feelings; provided acceptance and a sense of belonging, pride and respect; provided the opportunity to reflect on their past experiences, different perspectives, and new ideas; ending and beginning new relationships fostered personal growth and self-awareness; and finally, relationships provided them with hope, knowing that they can always turn to a family member or friends for support.

The significance of relationships positively impacting mental health and well-being is not a new concept. However, given the history of colonization and especially the residential school legacy, it is essential to understand the increased significance of relationships on contemporary Indigenous peoples’ mental health and well-being.

### Spiritual beliefs and cultural practices

Throughout the interviews, the néhiyawak spoke numerous times about the importance of their spiritual beliefs and cultural practices in improving their mental health, and essential for attaining optimal mental health and well-being. Their spiritual beliefs and participation in their cultural activities may be likened to enculturation as described by Zimmerman et al. [64]. They define *enculturation* as “the extent to which individuals identify with their ethnic culture, feel a sense of pride in their cultural heritage, and participate in traditional cultural activities” (p. 296). Ross [52] spoke about traditional ceremonies providing a culturally acceptable venue for Indigenous peoples to express their feelings. The néhiyawak said that their spirituality, praying, participating in cultural events such as a sweat, sundance, and pow wows improved their mental health and well-being.

One woman shared how her spirituality was making a positive difference to her mental health. She explained how spirituality had been integrated into her daily life and that to her was religion. She wondered how others cope without having spiritual connections and expressed empathy for those who attended the residential schools and were forbidden to practice their spirituality and culture:

My spirituality was integrated into my daily life. For example, if you go pick sweet grass, don’t pick in excess, only pick what you need, and make sure you put tobacco there for Mother Earth. That is religion to me; you’re connecting with your spirituality that way. The Creator is that close, intertwined in your life, that’s how I feel. I’m not religious. I see other young people whose parents went to residential school [and] when those parents came home, they didn’t go to church and they didn’t practice Indian culture. I often … wonder when they have hard times or when they struggle, how do *they* go forward to the next day, to the next year, without having that spiritual connection.

One male elder shared his healing journey involved exploring his identity as a Cree man:

Really, my mind was confused, my way of life was confused…in time I met an Elder and found out that I had to find my identity, who I really was as a Cree person. I needed to find out about my morals, my beliefs and my values as a Cree person. Which I did!

Colonization has disrupted Indigenous peoples’ spiritual beliefs and cultural practices [8, 43, 52]. It was only in 1933 that Indigenous peoples in Canada were allowed to participate in their traditional cultural practices without legal repercussions [44]. However, in recent decades, there has been a revival of Indigenous strength and determination across Canada driven by the restoration of traditional systems of beliefs and cultural practices, the recovery and reclamation of languages, the growth of First Nations’ sense of national identity, and the reconstruction and deconstruction of Aboriginal people’s history [62]. Today more Indigenous peoples are participating and finding renewed strength and spiritual resolve from participating in cultural ceremonies [11]. According to Kirmayer and Valaskakis [34], the recovery of tradition may be healing both at individual and collective levels. They assert restoring linguistic, religious, and communal practices as fundamental acts of healing. In addition, cultural ceremonies provide “individuals, families, and communities structures within which to acknowledge and mourn common wounds. Group healing, within ceremonies, reduces isolation; alleviates guilt, shame, and anger; and enhances feelings of self worth” [42].

Aboriginal cultures are rich with ceremonies designed to build strength, restore balance, and promote healing [8]. The néhiyawak narratives demonstrated their perceived benefits from attending cultural ceremonies. They described how, by attending and participating in the cultural and festive ceremonies, a cleansing of their mind, body, and soul occurred; this enabled them to have a “clear mind” and provided focus in their lives. The recovery of traditional ceremonies and practices engage individuals, families, and communities in ways that can promote solidarity, social support, and collective transformation [33].

### Tānisīsi wāpahtaman pimātisiwin (worldview)

This Cree phrase, *tānisīsi wāpahtaman pimātisiwin*, when translated means *worldview, how you see life, how you see the whole piece of life.* The néhiyawak in this study described how their worldview was influenced by their past experiences, formal, and informal education. The néhiyawak spoke about three primary areas that affected their tānisīsi wāpahtaman pimātisiwin (worldview) that improved their mental health and well-being: (a) taking personal responsibility, (b) their attitude, and (c) *wícihisowin*. This Cree phrase, wícihisowin, means *helping oneself*.

A male Elder shared how taking personal responsibility to make the changes in his life, attending Alcoholics Anonymous (AA), dealing with his anger, addressing his low self-esteem, focusing on positive thoughts, and having therapists who believed in him contributed positively to his mental health and well-being. He voiced, “what helped me is to really work hard on my problems, to listen, to practice, and to make changes in my life. Not just talking about it, but to make that change!” He acknowledged that he “couldn’t drink, be with people that use, or be in high-risk places such as bars or parties.” In addition, he stated he had “to deal with my anger, my resentment toward people who had hurt me.”

The néhiyawak explained that their attitude made a difference to their mental health and well-being. They described appreciating every day, thinking positively, accepting things as they are—believing there will be a positive outcome, having mutual respect, helping others, and having hope to improve their mental health. One young woman asserted, “just living every day to the fullest!” improved her mental health and well-being. A female Elder stressed the importance of being positive all the time and not worrying about the gossip. She was adamant in her belief that people often create their own stressors as a result of their lifestyle choices. She asserted:

A lot of people create their problems, because their lives or stories catch up with them. So, it’s best to be honest with yourself… It doesn’t matter how difficult, or negative a story is, I [change] it to something positive. I don’t focus and concentrate on the negative because I know that’s what causes stress and for people to become depressed. It doesn’t matter what other people say or think about you. What really matters, is how you feel about yourself!

A middle-aged man improved his mental health and well-being by having a positive attitude, working hard to achieve goals, and engaging in daily self-reflection. In addition, he stressed the importance of abstinence from drugs and alcohol made a difference to his mental health. He said:

It’s the way I look at life…be positive. You have got to work hard at everything you do. Anything you want, work hard for it… I don’t wake up in the morning feeling negative. This was passed on to me by my grandfather, and my parents, be happy when you wake up every morning, somewhere along the way things might change, but at least start your day off right. In my house, I don’t allow anybody to be grumpy, or mad, in the morning, I just say, ‘not now, don’t do that now, it’s the morning, let’s get up happy.’ And you never give up, you never ever give up!… It is important to abstain from drugs and alcohol because it never was a part of our culture and it doesn’t fit in our culture. I’ve never done drugs of any kind, including pain killers.

McCormick [39] found setting goals facilitated healing for the First Nations people of British Columbia, they felt more optimistic and empowered. Examples ranged from setting career goals to goals of improving one aspect of one’s life. Before setting goals many of his participants reported feeling depressed and powerless due to lack of options and direction. Hart [20] explained that it is “through the taking of responsibility for their own personal healing and growth that individuals will be able to attain *mino*-*pimatisiwin*,” meaning “the good life” (p. 44). Mussell [43] described healing to begin with a personal decision, deciding that it is time to find help and to take the necessary risks involved in healing; often beginning by talking and expressing the feelings they have kept inside. Interestingly, in the Cree language there is one word that describes taking responsibility for all aspects of one’s life, *manācihisowin*. When translated, it means *a person’s responsibility to look after all aspects of their lives. This entails living a clean lifestyle, physically, emotionally and spiritually.*

Wícihisowin was achieved by the néhiyawak participating in formal and informal educational opportunities. One man talked about taking the training to be a life skills coach improved his mental health and well-being. As a result of the training, he was reacting differently to his environment and had changed his lifestyle. He said, “For myself, it is having that ability to … notice things and react differently to situations. I’m a life skills coach; it helped me a lot personally to adjust myself and my lifestyle.”

One very young woman attributed her past experiences, environment, and her children as her inspiration to make changes and improve her mental health. She spoke about the difficulty of living in an area that was “surrounded by alcoholics.” When she was incarcerated she continued to be surrounded by negativity. Thus, these life experiences provided the impetus for her to change the direction of her life. She talked about the importance of the courses she completed while incarcerated. These courses helped her to understand and manage her emotions, addressed relationships, and parenting. She spoke with enthusiasm about graduating this year, making new friends, and celebrating two years of abstinence from marijuana. She articulated:

Just seeing everything around me! I live in the town site, and it’s nothing but alcoholics. I don’t want to live that way; I don’t want to end up living like that. My kids! I don’t want to smoke anymore. I want to watch them have kids and be there to support them. I actually just got out of jail, being in there and seeing all that negative stuff was like, ‘whoa.’ It really was an eye opener! I took a bunch of classes in jail that were very helpful. I took emotions management, relationship skills, and two parenting classes. The classes were all for self-help…my schooling, I’m doing my adult 12 this year. I actually quit smoking up for almost 2 years now. Yeah! I lost some friends, but obviously they weren’t friends. I made new friends. I’m not such a burnout [laughs] anymore.

Overall, the concepts described within tānisīsi wāpahtaman pimātisiwin (taking personal responsibility, attitude, and wícihisowin) correlate with eight of the 14 categories known to facilitate First Nations healing as described in McCormick’s study [39]. McCormick found the following categories facilitated healing for the First Nations people of British Columbia: (a) exercise; (b) involvement in challenging activities; (c) expressing oneself; (d) setting goals; (e) helping others; (f) gaining an understanding of the problem; (g) learning from a role model; and, (h) establishing a connection with nature. The vast majority of the néhiyawak in this study were already engaged in these *healing activities* as described by McCormick [39].

Within this theme, tānisīsi wāpahtaman pimātisiwin, the néhiyawak voiced several concepts related to their worldviews, the lens through which they view the world. They described how taking personal responsibility, their attitude, and wícihisowin improved their mental health and well-being. Personal responsibility entailed practicing healthy communication patterns; making the required changes in their life; acknowledging, addressing, and dealing with their emotions and feelings; having healthy boundaries; and exercising choice. Having a positive attitude made a difference to their mental health and well-being. A positive attitude included: appreciating every day; thinking positive at all times; abstaining from drugs and alcohol; practicing acceptance and respect; helping others; having hope; and a belief in an afterlife. wícihisowin, “helping oneself,” was achieved by participation in both formal and informal educational opportunities.

### Ēkwa ōhi kikwaya piko ka-ispayiki kīspin ka-nohtē-miyo-mahcihoyān (these are the things that need to happen if I want to be healthy)

The Cree phrase, *ēkwa ōhi kikwaya piko ka*-*ispayiki kīspin ka*-*nohtē*-*miyo*-*mahcihoyān*, translates to *these are the things that need to happen if I want to be healthy.* The one word in Cree, *miyo*-*mahcihoyān*, takes into account the physical, emotional, mental, and spiritual well-being of an individual. When the néhiyawak were asked what they needed to attain optimal mental health and well-being they emphatically reiterated the importance of their relationships, spiritual beliefs and cultural practices, and their tānisīsi wāpahtaman pimātisiwin; however, there was an increased emphasis on aspects related to their socio-economic status, elements described by both the medicine wheel [43] and the determinants of health [47].

Indigenous knowledge provides the philosophical foundation for the medicine wheel. Although the medicine wheel symbol has different meanings and expressions for different First Nations, some of the principles are universal [1, 55]. Sevenson and Lafontaine [55] affirmed “that everything is related to everything else, that things cannot be understood outside of their context and interactions, and that there are four aspects to the human condition-the physical, the emotional, the mental and the spiritual” (p. 190). Mussell [43] asserted that an individual’s physical, emotional, mental, and spiritual needs must be met for the development of human potential and “are required for survival and personal growth” (p. 115).

However, several of the néhiyawak in this study expressed that they were content with their current state of mental health and well-being and did not need anything else to attain optimal mental health and well-being. One female Elder responded, “I consider myself strong, healthy, as possible. I’ve did the best that I can.” Another very young lady asserted, “Right now nothing… I just like the way it’s going right now, I just like it… I love it, you caught me at a time where I’m thriving.” One middle-aged man felt that he would be able to manage whatever challenges the future may hold:

I think I’m in that place where I have that ability to change with whatever comes good or bad; it’s up to me. I know a lot of times it hurts and at some point you have to stand up and say, ‘Ok, now I have to go on.’ I don’t need to see a shrink or anything. Well, at one point I thought of it, but would never do it. Really it is self-pity; it’s just self-pity that you come out of, I do anyway. I just laugh [he laughed] and say, ‘Ok big baby, life goes on.’

The remaining néhiyawak clearly articulated what they required to attain optimal mental health and well-being. They voiced physical and intellectual (mental) needs that correspond with the medicine wheel [43] and the determinants of health [47].

### The néhiyawak physical needs

The vast majority of the néhiyawak spoke of physical needs as described by both the medicine wheel [43] and/or by two determinants of health—(a) employment/working conditions, and (b) income and social status [47]. The néhiyawak physical needs were related to securing employment; having a home, a safe environment to live in; being able to buy sufficient nutritious food; being able to manage their chronic health conditions; and exercising on a regular basis. They saw the meeting of these physical needs as necessary to attain optimal mental health and well-being.

Numerous néhiyawak mentioned that being employed made a difference to their mental health and well-being and employment was perceived as necessary to attain optimal mental health and well-being. They described employment as providing stimulation, giving them hope, providing income which increased their personal freedom, and providing structure for their lives. One young woman spoke about how having a job prevented boredom and, more importantly, provided her with stimulation: “working, just keeping busy all the time, not being bored.” Another young woman described how her approaching graduation and the anticipation of obtaining employment gave her hope and made a difference to her mental health and well-being. She said, “I hope to graduate from university soon… hope to get a good job.” Another woman articulated how she had worked most of her life, she enjoyed her job, and most importantly the income allowed her more personal freedom. She asserted, “I’ve worked most of my life. I just enjoy working, I get along with people. Keeps me busy, I don’t get bored and [with] the money… I can do more.”

The ability for the néhiyawak to meet their physical needs is related to their income, whether or not they have employment. Income determines an individual’s living conditions, such as safe housing or whether they will be able to buy sufficient healthy food [47]. Health status improves at each step up the income and social hierarchy, and there is mounting evidence that higher socio-economic status is associated with better health. In fact, these two factors (income and social status) seem to be the most important determinants of health [47, 49] from a Western perspective.

Another young woman improved her mental health and well-being by exercising regularly, she shared “[Being] active, exercising. I go to the track in the evenings.” In McCormick’s [39] study of facilitating healing for First Nations people in British Columbia, he found exercise helped his participants to feel better about themselves because they were able to feel stronger and more capable. We have known for a long time about the benefits of exercise as a proactive way to enhance our physical condition and combat disease; now, exercise is recognized as an essential element in building and maintaining mental fitness [6].

### The néhiyawak intellectual/mental needs

According to the medicine wheel as described by Mussell [43], the intellectual/mental needs encompass an individual’s ideas, concepts, thoughts, habits, and discipline; all the mental tools used to make sense of, and to cope with, life. Numerous néhiyawak in this study thought it was necessary to abstain from drugs and alcohol, and this was perceived as necessary for them to attain optimal mental health and well-being.

One middle-aged woman said that she didn’t want to die as a statistic related to alcohol abuse even though both her parents did and she feared her biological siblings would die in the same manner. In addition, she said that she would have to change her social circle of friends to include non-drinkers. She proclaimed:

I don’t want to die as a statistic from alcohol or from abuse. Both of my biological parents had passed away that way. Even to this day my biological sisters and my brother, I think that they’re going to die from alcohol abuse. I just don’t want to be like that.

According to the First Nations Regional Longitudinal Health Survey [14], community wellness is dependent on some form of sobriety. Sinclair et al. [56] identified mental health and addictions as the most significant health issues for many Métis and First Nations communities. Their study also noted these issues were related to the larger social determinants of health, particularly poverty, and lack of education and employment opportunities.

Several of the néhiyawak in this study identified the importance of continuing their education as being necessary for them to obtain optimal mental health and well-being. One very young man who was currently in grade 12 said, “School, just having a feeling of going there every day … something to do … nothing bugs me there.” Another very young man was emphatic that he and his spouse required careers in order for him to achieve optimal mental health and well-being. He declared, “we would both have to have a career… *we need* to have careers, not *I want.*”

Education and literacy is a determinant of health, and health status improves with an individual’s level of education [47]. Education is closely connected to an individual’s socio-economic status, and effective education for children and lifelong learning for adults are key contributors to health and prosperity for individuals, and for the country. It also contributes to health and prosperity by equipping people with knowledge and skills for problem solving and helps provide a sense of control and mastery over life circumstances. Education increases opportunities for job and income security, and job satisfaction. It improves people’s ability to access and understand information about healthy lifestyles [36, 47]. According to Statistics Canada [59], Aboriginal adults consistently have lower graduation rates than other Canadians. When comparing the attainment of a university degree, only 9.8 % of Aboriginal people have a degree compared to 26.5 % of non-Aboriginal Canadians.

There are many factors that can influence one’s health, including one’s mental health. These factors are commonly known as the determinants of health [23, 47]. It has become increasingly accepted that health is interdependent on a variety of factors [41, 47, 48]. Raphael [48] explained that the social determinants of health are the economic and social conditions that influence the health of individuals and communities. The economic and social conditions of a person’s life have “far greater influence on health and the incidence of illness than traditional biomedical and behavioural risk factors” (p. 2).

The second goal in *A Framework for a Mental Health Strategy for Canada* [41], acknowledged the need to attend to the complex interaction of economic, social, psychological, and biological or genetic factors that is known to determine mental health and mental illness across the lifespan. Within this goal there is reference to address adequate housing, and to reduce whenever possible those factors that increase the risk of developing mental health problems and illnesses, such as poverty, abuse, and social isolation [41].

Irrefutably, there are unacceptable disparities between the economic and social conditions of Indigenous people and non-Indigenous people in Canada [22, 58]. The four themes derived from this narrative inquiry correspond to six of the determinants of health: income and social status; social support networks; education and literacy; employment; personal health practices and coping skills; and culture [47]. Socio-economic status has been cited as the most important determinant of health [36, 48] and without dispute, this determinant was disrupted by colonization [45, 62].

In a previous study [18], the perceptions of health from a Plains Cree perspective were explored and findings supported the determinants of health as an appropriate framework to address the health needs of Indigenous peoples and as a framework for federal, provincial, and local policy makers to implement structural changes necessary to decrease the health disparities between the Indigenous peoples and the rest of Canada. Using a consistent framework, such as the determinants of health, is useful to provide a benchmark from which to evaluate existing disparities, initiate changes in existing policy and program implementation, and to measure and evaluate improvement.

## Discussion

When the néhiyawak were asked what was making a difference for them in terms of their mental health, they responded by sharing concepts and values that are inherent within Indigenous worldviews and they mentioned the necessity of self-sufficiency as making a difference for their mental health and required for them to obtain optimal mental health and well-being. Given the historical and current context of colonization, it is not surprising that the néhiyawak in this study identified and are using the same concepts and values to improve their mental health and well-being that were disrupted and in some cases destroyed by colonization [8, 43, 62]. In fact, they considered these concepts and values essential for them to attain optimal mental health and well-being.

Interestingly, the themes derived from this study appear analogous with Indigenous worldviews that have been described by various Indigenous and non-Indigenous scholars. McCormick [40] described traditional Aboriginal worldviews to include: “balance, connectedness, spirituality, nature, ceremony, and culture” (p. 357). Hart [20] described Aboriginal worldviews to include: wholeness, balance, harmony and described the key supporting values to be: respect, caring, faith, honesty, kindness, and sharing. Epes-Brown [11] described core Indigenous values and perspectives to include: interconnectedness, reciprocity, language, and ceremony. The Indigenous concepts and values shared by these authors were voiced and reiterated numerous times throughout the néhiyawak narratives.

The findings from this study were organized and presented as separate themes; yet from an Indigenous worldview, one can see the interconnectedness and relationships between the themes and the relational concepts inherent within the teachings of the medicine wheel. These four themes, as described by the néhiyawak, have elements that parallel the needs for development of human potential as described by the medicine wheel [43] and the determinants of health [47].

Connectedness is fundamental to Indigenous worldviews and colonization was the beginning of “dis-connection” [8], p. 44). However, despite the legacy of colonization, the néhiyawak in this study were relatively content with their mental health and well-being. It is interesting that asking different questions—focusing on strengths as opposed to deficits—allowed the néhiyawak to shine, bringing their strengths and resilience to the forefront. Asking different questions will result in different answers. The results from this study show the vast majority of the néhiyawak in this study were connected to their families, spiritual beliefs, and cultural practices in contrast to the disconnection described by Chansonneuve [8] that has plagued Indigenous peoples since European contact.

## Conclusions

Recognizing that Thunderchild is only one of many First Nations communities, this research represents a contribution to the current body of knowledge addressing néhiyawak miyo-mahcihoyān (well-being). The insight gathered from an Indigenous perspective is essential for planning effective health promotion for Indigenous populations [4, 12, 16, 19, 22]. Most importantly, this inquiry highlights a contemporary Indigenous voice within Thunderchild First Nation on what is making a positive difference to their mental health and what they perceive as necessary to attain optimal mental health and well-being.

Despite the resilience of the néhiyawak in this study, the current statistics portray unacceptable health disparities between the Indigenous and non-Indigenous peoples of Canada [22, 38, 58, 60]. Determinants of health, especially income and education, require immediate attention to allow Indigenous peoples an opportunity to attain the same level of health as non-Indigenous people in Canada. Levels of income, employment, education, housing, health, and mental health vary considerably between Indigenous and non-Indigenous people in Canada [22, 26, 58]. It is time to restore the imbalance that was created with colonization, to collaborate and expeditiously implement the structural changes required to address the existing health disparities between the Indigenous peoples and non-Indigenous peoples of Canada. Ideally, this endeavor would involve joint collaboration between all levels of government, both Indigenous and non-Indigenous. Ultimately, improving the health of Canada’s Indigenous peoples will depend on improving their economic and social conditions as well as assisting Indigenous peoples to identify and address their own health needs.

## Abbreviations

AA: Alcoholics Anonymous
BCR: Band Council Resolution
CMA: Canadian Health Mental Association
FNC: First Nations Centre
FNIGC: First Nations Information Governance Centre
FNRHS: First Nations Regional Health Survey
OTC: Office of the Treaty Commission
PHAC: Public Health Agency of Canada
TCPS2: Tri-Council Policy Statement

## References

[1] Absolon K (1993). Healing as Practice: Teachings from the medicine wheel. Commissioned paper for the Wunska Network the Canadian Association of Schools of Social Work.

[2] Adelson N, Kirmayer LJ, Valaskakis GG (2009). Cultural continuity as a moderator of suicide risk among Canada’s First Nations. Healing traditions: the mental health of Aboriginal peoples in Canada.

[3] Antone RA, Hill DL, Meyers BA (1986). The power within people.

[4] Bartlett J (2005). Health and well-being for Métis women in Manitoba. Can J Public Health.

[5] Canadian Mental Health Association. Definition of mental health and wellness. Ottawa: Author; 2009. http://www.cmha.ca/bins/content_page.asp?cid=2-267-1319&lang=1. Accessed 15 Jan 2011.

[6] Canadian Mental Health Association. Get physical. Ottawa: Author; 2011. http://www.cmha.ca/bins/content_page.asp?cid=2-267-1320-1321&lang=1. Accessed 15 Jan 2011.

[7] Caron NR (2005). Getting to the root trauma in Canada’s Aboriginal population. Can Med Assoc J.

[8] Chansonneuve D (2005). Reclaiming connections: understanding residential school trauma among Aboriginal people.

[9] Clandidin DJ, Connelly FM (2000). Narrative inquiry: experience and story in qualitative research.

[10] Creswell JW, Miller DL (2000). Determining validity in qualitative inquiry. Theory Pract.

[11] Epes-Brown J, Dooling DM, Jordon-Smith P (1992). Becoming part of it. I became a part of it: sacred dimensions in Native American life.

[12] Ermine W, Sinclair R, Jeffery B (2004). The ethics of research involving Indigenous peoples: report of the Indigenous peoples’ health research centre to the interagency advisory panel on research ethics (PRE).

[13] First Nations Centre. OCAP: ownership, control, access and possession. Sanctioned by the First Nations Information Governance Committee, Assembly of First Nations. Ottawa: National Aboriginal Health Organization. 2007. http://cahr.uvic.ca/nearbc/documents/2009/FNC-OCAP.pdf. Accessed 15 Jan 2011.

[14] First Nations Centre. First nations regional longitudinal health survey (RHS) 2002/3. Ottawa: FNC; 2005. http://fnigc.ca/sites/default/files/ENpdf/RHS_2002/rhs2002-03-technical_report.pdf. Accessed 15 Jan 2011.

[15] First Nations Information Governance Centre (FNIGC). First nations regional health survey (RHS) 2008/10: National report on adults, youth and children living in First Nations communities. Ottawa: FNIGC; 2012. http://fnigc.ca/sites/default/files/docs/first_nations_regional_health_survey_rhs_2008-10_-_national_report.pdf. Accessed 15 Jan 2011.

[16] Fishbein M, Ajzen I (1975). Belief, attitude, intention, and behavior: an introduction to theory and research.

[17] Gillis A, Jackson W (2002). Research for nurses: methods and interpretation.

[18] Graham H, Stamler LL (2010). Contemporary perceptions of health from an Indigenous (Plains Cree) perspective. J Aborig Health.

[19] Hakim H, Wegmann DJ (2002). A comparative evaluation of the perspectives of health of elders of different multicultural backgrounds. J Community Health Nurs.

[20] Hart MA (2002). Seeking mino-pimatisiwin: an Aboriginal approach to helping.

[21] Health Canada. Health policy research bulletin, vol 1, issue 5. Ottawa. Author; 2003. http://www.hc-sc.gc.ca/iacb-dgiac/arad-draa/english/rmdd/bulletin/aboriginal.html#page6. Accessed 6 Sep 2016 **(Health status)**.

[22] Health Canada. Closing the gaps in Aboriginal health. Ottawa, ON: Author; 2005. Retrieved 6 Sep 2016 from http://www.hc-sc.gc.ca/sr-sr/pubs/hpr-rpms/bull/2003-5-aboriginal-autochtone/index-eng.php.

[23] Health Canada. First Nations, Inuit and Aboriginal Health. 2007. http://www.hc-sc.gc.ca/sr-sr/pubs/hpr-rpms/bull/2003-5-aboriginal-autochtone/index-eng.php#ab1. Accessed 15 May 2016.

[24] Health Canada. Statistical profile on the health of First Nations in Canada. 2009. http://www.hc-sc.gc.ca/fniah-spnia/pubs/aborig-autoch/2009-stats-profil/index-eng.php. Accessed 15 May 2016.

[25] Health Canada. A statistical profile on the health of First Nations in Canada: vital statistics for Atlantic and Western Canada. Ottawa: Author; 2014. http://www.hc-sc.gc.ca/fniah-spnia/pubs/aborig-autoch/2007-stats-profil-vital-demo/index-eng.php. Accessed 6 Sep 2016.

[26] Health Council of Canada. The health status of Canada’s First Nations, Métis, and Inuit people: a background paper to accompany. Health Care Renewal in Canada: accelerated change. Ottawa: Author; 2005. http://healthcouncilcanada.ca/docs/papers/2005/BkgrdHealthyCdnsENG.pdf. Accessed 15 Jan 2011.

[27] Hill BH (2002). Shaking the rattle: healing the trauma of colonization.

[28] Khan S (2008). Aboriginal mental health: the statistical reality. Vis J.

[29] Karmali S, Laupland K, Harrop R, Findlay C, Kirkpatrick AW, Winston B, Hameed M (2005). Epidemiology of severe trauma among status Aboriginal Canadians: a population-based study. Can Med Assoc J.

[30] Kielland N, Simeone T. Current Issues in Mental Health in Canada: The Mental Health of First Nations and Inuit Communities. Ottawa: Parliament of Canada, Legal and Social Affairs Division; 2014. http://www.lop.parl.gc.ca/content/lop/ResearchPublications/2014-02-e.htm#a3. Accessed 6 Sep 2016.

[31] Kirmayer LJ, Brass GM, Holton T, Paul K, Simpson C, Tait C (2007). Suicide among Aboriginal people in Canada.

[32] Kirmayer LJ, Brass GM, Tait CL. The mental health of Aboriginal peoples. In: Kirmayer LJ, Macdonald ME, Brass GM, editors. Proceedings of the Advanced Study Institute: The Mental Health of Indigenous Peoples. Montreal: McGill Summer Program in Social & Cultural Psychiatry and the Aboriginal Mental Health Research Team; 2000. https://www.mcgill.ca/tcpsych/files/tcpsych/Report10.pdf. Accessed 15 Jan 2011.

[33] Kirmayer LJ, Brass GM, Valaskakis GG, Kirmayer LJ, Valaskakis GG (2009). Conclusion: healing/intervention/tradition. Healing traditions: the mental health of Aboriginal peoples in Canada.

[34] Kirmayer LJ, Valaskakis GG, Kirmayer LJ, Valaskakis GG (2007). Preface. Healing traditions: the mental health of Aboriginal peoples in Canada.

[35] Kovach M (2009). Indigenous methodologies: characteristics, conversations, and contexts.

[36] Labonte R (2003). How our programs affect population health determinants: a workbook for better planning and accountability.

[37] Lincoln YS, Guba EG (1985). Naturalistic inquiry.

[38] MacMillan H, MacMillan A, Offord D, Dingle J (1996). Aboriginal health. Can Med Assoc.

[39] McCormick R (1995). The facilitation of healing for the First Nations people of British Columbia. Can J Nativ Educ.

[40] McCormick R, Kirmayer L, Valaskakis GG (2009). Aboriginal approaches to Counselling. Healing traditions: the mental health of Aboriginal peoples in Canada.

[41] Mental Health Commission of Canada. Toward recovery & well-being: a framework for a mental health strategy for Canada. Ottawa: Author; 2009. http://www.mentalhealthcommission.ca/English/document/241/toward-recovery-and-well-being. Accessed 6 Sep 2016.

[42] Mitchell TL, Maracle DT (2005). Healing the generations: post-traumatic stress and the health status of Aboriginal populations in Canada. J Aborig Health.

[43] Mussell WJ (2005). Warrior-caregivers: understanding the challenges and healing of First Nations men.

[44] Office of the Treaty Commissioner (2008). Treaty essential learnings: we are all treaty people.

[45] Paul DN (2006). First Nations history: We were not the savages.

[46] Public Health Agency of Canada. The human face of mental health and mental illness in Canada. Ottawa: Author; 2006. http://www.phac-aspc.gc.ca/publicat/human-humain06/3-eng.php. Accessed 6 Sep 2016.

[47] Public Health Agency of Canada. What determines health? 2010. http://www.phac-aspc.gc.ca/ph-sp/determinants/index-eng.php. Accessed 6 Sep 2016.

[48] Raphael D, Raphael D (2004). Introduction to the social determinants of health. Social determinants of health: Canadian perspectives.

[49] Raphael D, Raphael D, Bryant T, Rioux M (2006). Social determinants of health: an overview of concepts and issues. Critical perspectives on health, illness, and health care: staying alive.

[50] Reissman CK (2008). Narrative methods for the human sciences.

[51] Roberts R (2005). Stories about cancer from the Woodland Cree of northern Saskatchewan.

[52] Ross R (1992). Dancing with a ghost: exploring Indian reality.

[53] Royal Commission on Aboriginal Peoples (1995). Choosing life: special report on suicide among Aboriginal people.

[54] Schnarch B (2004). Ownership, control, access, and possession (OCAP) or self-determination applied to research: a critical analysis of contemporary First Nations research and some options for First Nation communities.

[55] Sevenson KA, Lafontaine C, First Nations and Inuit Regional Health Survey Steering Committee (2003). Chapter six: the search for wellness. First Nations and Inuit Regional Health Survey.

[56] Sinclair R, Smith R, Stevenson N (2006). Miyo-māhcihowin: a report on Indigenous health in Saskatchewan.

[57] Smith LT (1999). Kaupapa Maori methodology: our power to define ourselves. A seminar presentation to the School of Education.

[58] Statistics Canada. Aboriginal statistics at a glance. Ottawa: Author; 2010. http://www.statcan.gc.ca/pub/89-645-x/89-645-x2010001-eng.htm. Accessed 15 May 2016.

[59] Statistics Canada. The educational attainment of Aboriginal peoples in Canada. Ottawa: Author; 2011. https://www12.statcan.gc.ca/nhs-enm/2011/as-sa/99-012-x/99-012-x2011003_3-eng.cfm. Accessed 6 Sep 2016.

[60] Tookenay VF (1996). Improving the health status of Aboriginal people in Canada: new directions, new responsibilities. Can Med Assoc.

[61] Waldram JB, Herring DA, Young TK (1995). Aboriginal health in Canada: historical, cultural, and epidemiological perspectives.

[62] Wesley-Esquimaux CC, Smolewski M (2004). Historic trauma and Aboriginal healing.

[63] Wheatley M (1996). The challenges of research in Native American communities: incorporating principles of cultural competence. J Soc Serv Res.

[64] Zimmerman MA, Ramirez-Valles J, Washienko KM, Walter B, Dyer S (1996). The development of a measure of enculturation for Native American youth. Am J Community Psychol.

